# Impact of two *Erwinia* sp. on the response of diverse *Pisum sativum* genotypes under salt stress

**DOI:** 10.1007/s12298-024-01419-8

**Published:** 2024-02-25

**Authors:** Houda Ilahi, Elisa Zampieri, Cristiana Sbrana, Francesca Brescia, Luca Giovannini, Roghayyeh Mahmoudi, Gholamreza Gohari, Mustapha Missbah El Idrissi, Mohamed Najib Alfeddy, Martino Schillaci, Lahcen Ouahmane, Alice Calvo, Fabiano Sillo, Vasileios Fotopoulos, Raffaella Balestrini, Bacem Mnasri

**Affiliations:** 1 Faculty of Sciences of Tunis, University Tunis El Manar, 2092 Tunis, Tunisia; 2 Laboratory of Legumes and Sustainable Agroecosystems, Centre of Biotechnology of Borj-Cédria, BP 901, 2050 Hammam-Lif, Tunisia; 3 Institute for Sustainable Plant Protection (IPSP), National Research Council of Italy, Strada Delle Cacce 73, 10135 Turin, Italy; 4 Institute of Agricultural Biology and Biotechnology (IBBA), National Research Council of Italy, Via Moruzzi 1, 56124 Pisa, Italy; 5 Department of Agricultural Sciences, Biotechnology and Food Science, Cyprus University of Technology, 3036 Limassol, Cyprus; 6 Faculty of Sciences, Centre de Biotechnologies Végétale et Microbienne, Biodiversité et Environnement, Mohammed V University in Rabat, Rabat, Morocco; 7 Phytobacteriology Laboratory Plant Protection Research, Unit CRRA Marrakesh National Institute for Agronomical Research Marrakesh, 40000 Marrakesh, Morocco; 8 Laboratory of Microbial Biotechnologies Agrosciences and Environment, Cadi Ayyad University, 40000 Marrakesh, Morocco

**Keywords:** Abiotic stress, Biochemical markers, Pea, 16S rRNA/DNA, RT-qPCR

## Abstract

Currently, salinization is impacting more than 50% of arable land, posing a significant challenge to agriculture globally. Salt causes osmotic and ionic stress, determining cell dehydration, ion homeostasis, and metabolic process alteration, thus negatively influencing plant development. A promising sustainable approach to improve plant tolerance to salinity is the use of plant growth-promoting bacteria (PGPB). This work aimed to characterize two bacterial strains, that have been isolated from pea root nodules, initially called PG1 and PG2, and assess their impact on growth, physiological, biochemical, and molecular parameters in three pea genotypes (Merveille de Kelvedon, Lincoln, Meraviglia d’Italia) under salinity. Bacterial strains were molecularly identified, and characterized by in vitro assays to evaluate the plant growth promoting abilities. Both strains were identified as *Erwinia* sp., demonstrating in vitro biosynthesis of IAA, ACC deaminase activity, as well as the capacity to grow in presence of NaCl and PEG. Considering the inoculation of plants, pea biometric parameters were unaffected by the presence of the bacteria, independently by the considered genotype. Conversely, the three pea genotypes differed in the regulation of antioxidant genes coding for catalase (*PsCAT*) and superoxide dismutase (*PsSOD*). The highest proline levels (212.88 μmol g^−1^) were detected in salt-stressed Lincoln plants inoculated with PG1, along with the up-regulation of *PsSOD* and *PsCAT*. Conversely, PG2 inoculation resulted in the lowest proline levels that were observed in Lincoln and Meraviglia d’Italia (35.39 and 23.67 μmol g^−1^, respectively). Overall, this study highlights the potential of these two strains as beneficial plant growth-promoting bacteria in saline environments, showing that their inoculation modulates responses in pea plants, affecting antioxidant gene expression and proline accumulation.

**Supplementary Information:**

The online version contains supplementary material available at 10.1007/s12298-024-01419-8.

## Introduction

Leguminous species such as pea (*Pisum sativum*) are an important source of nutritional components due to the seed richness in proteins, carbohydrates, vitamins, minerals, fibers and antioxidant compounds (Sapre et al. 2022). Pea can be grown in different regions, and it is fourth in legume global production after soybean, peanut, and dry beans (Vidal-Valverde et al. 2003). It is an important food and fodder legume for Mediterranean countries, including Tunisia, due to high nutritional value of their protein and starch-rich seeds (El Idrissi et al. 2020). Being a legume species, pea cultivation increases the content of nitrogenous compounds in nitrogen-poor soils (Kebede 2021). However, throughout the course of the five years from 2017 to 2021, pea output worldwide decreased by around 10% as a result of declining soil productivity and harvested area overall (FAOSTAT 2021). This scenario will be exacerbated by the increase of food demand (from 35 to 56%) globally, and the subsequent need to achieve increased crop yields under the world growing population (van Dijk et al. 2021). Addressing this challenge needs effective agricultural management to both enhance crop resilience to abiotic stresses and to preserve soil fertility and health, especially under environmental constraints (van Dijk et al. 2021).

Arid and semi-arid areas are among the most vulnerable to environmental constraints that are exacerbated by climate change. Salinity is a relevant environmental limiting element, causing major reductions in plant productivity (Peng et al. 2023; Raza et al. 2022). It is estimated that more than 50% of the arable soils in the world will be salinized by 2050 (Etesami et al. 2022). In Tunisia, about 25% of the cultivated area is affected by salinity, and it is evaluated that 9.13% of the total Tunisian surface is challenged by this threat due to aridity and poor water management (Aloui et al. 2022). The growth of crops is negatively affected by salt stress, which causes osmotic stress due to decreased water availability and ion toxicity from nutritional imbalances (Acosta-Motos et al. 2017).

Although *P. sativum* is recognized to be moderately tolerant to soil salinity (Shahid et al. 2022), elevated salt levels in soil were reported to significantly affect its productivity (Noreen and Ashraf 2009). In previous few years, inoculation of plants with beneficial soil microbes, including plant growth-promoting bacteria (PGPB), has proven to be a useful approach to enhance sustainable agriculture in soils subjected to several abiotic stresses, including salinity (Bhat et al. 2023, 2020; Peng et al. 2023; Mishra et al. 2021). They can increment the germination of seeds as well as the leaf size, improve chlorophyll and protein amount, increase crop growth and yield, in addition to nutrient accessibility, and delay the senescence of leaves (Saghafi et al. 2019). They ameliorate salt stress tolerance by different mechanisms, *i.e.*, synthetizing antioxidant enzymes, non-enzymatic antioxidants, and osmolytes (*e.g.*, proline), improving nutrient uptake, producing 1-aminocyclopropane-1-carboxylate (ACC) deaminase, indole acetic acid (IAA), siderophores and exopolysaccharide (EPS; Peng et al. 2023; Saghafi et al. 2019). Endophytic bacteria, such as *Bacillus subtilis* and *Pseudomonas fluorescens*, significantly decreased Na^+^ accumulation and promoted K^+^ uptake in pea under salt stress, affecting osmoregulation and antioxidant capacity (Sofy et al. 2021). Plant salt tolerance can be affected by the capacity of microbes to regulate the expression of plant transcription factors involved in stress responses, additionally to the synthesis of enzymes correlated to reactive oxygen species (ROS), proline and the synthesis of EPS (Aeron et al. 2020). It was observed that PGPB may improve plant development in a way that depends on the bacterial strain and plant genotype, as different plant genotypes may differentially react to the same microbial strain (Wei and Jousset 2017). Several reports have shown the potential of PGPB in improving productivity of many legume plants, including peanuts (Sharma et al. 2016), *Sulla carnosa* (Hidri et al. 2016) and *Lathyrus cicera* (Gritli et al. 2022) under salt stress conditions. Inoculation with *Planomicrobium* sp. MSSA-10 significantly increased pea growth under salt stress, decreasing ROS and enhancing antioxidant enzyme activities (Shahid et al. 2018). *Variovorax paradoxus* 5C-2 has been found to mitigate salt stress in pea, enhancing water relations, ion homeostasis and photosynthesis (Wang et al. 2016), while the application of *P. sativum* with *Bacillus marisflavi* (CHR JH 203) and *Bacillus cereus* (BST YS1_42) affected the expression of genes involved in ROS scavenging, defense and cell rescue, and enhanced growth and tolerance to the stress itself in greenhouse under salt stress (Gupta et al. 2021a).

Several bacterial species have been characterized as PGPB, including species belonging to genera known to be plant pathogens (Wei et al. 2023; Passera et al 2019). Among them, *Pseudomonas syringae* is a bacterial genus that include strains with beneficial effect on plants (*i.e., P. syringae* pv. *syringae* strain 260–02) and others that, on the contrary, are pathogenic ones (*i.e., P. syringae* pv. tomato strain DC3000) (Passera et al. 2019). It is known that *Erwinia* species may cause fire blight to Rosaceae plants (*e.g.*, *E. amylovora*), and bacterial blight to cucurbits and other dicotyledonous plants, soft rot in a broad-host-range (*E. carotovora*), but also show positive associations with plants such as tea, rice and wheat (*e.g.*, *E. tasmaniensis* and *E. billingiae*) (Jia et al. 2022; Sagar et al. 2018). The application of *Erwinia* sp. as PGPB significantly improved tomato fruit fresh and dry weight and yield compared with *Bacillus pumilus* and *P. putida* (Shen et al. 2012).

This study was aimed to verify the impact of two strains of *Erwinia* sp., previously isolated from pea root nodules, on alleviating salt stress in three *P. sativum* genotypes, using both two cultivars largely employed in Tunisia (Merveille de Kelvedon and Lincoln) and one dwarf genotype commercialized in Italy (Meraviglia d’Italia). The effects of the bacterial inoculation on pea response to a salt stress condition were evaluated by considering biometric parameters, biochemical traits, and expression of genes previously correlated with pea salt stress response.

## Materials and Methods

### Isolation of the bacterial strains

Soil of two fields in Tunisia served as the source of the pea nodule associated bacterial strains. Briefly pea plants (Merveille de Kelvedon cultivar) were grown using soil samples from two northern Tunisian locations (Mograne and Zaghwan) for capturing bacteria found in the native soil (Ilahi et al. 2021). For each region, three soil samples were collected at a distance of 50 m apart from each other and they were mixed to obtain a pooled sample by location. Pea seeds were sterilized and germinated as described by Gritli et al. (2019). The seedlings were grown in a greenhouse for fifty days upon the natural light with a daily temperature of 20–24 °C (minimum–maximum). At the end of the growing period, root nodules were collected and sterilized on the surface according to Vincent's standard procedures (Mnasri et al. 2009). Bacterial strains were isolated from collected root nodules by selecting individual colonies on mannitol yeast agar (YEMA) (Vincent 1970). The isolation of 20 isolates, grown on YEMA-RC (Red Congo) medium at 28 °C, allowed to distinguish two different groups of bacterial strains. The first majority group (16/20) was composed of bacteria with slow growth (appearance of colonies after four days). The second minority group (4/20) included fast-growing bacteria (appearance of colonies after two days). Two strains (called PG1 and PG2), belonging to the second group (*i.e.*, both showing a fast growth), were selected for biochemical and molecular characterizations.

### Estimation of PGP activities in vitro

Different biochemical capabilities have been assessed in PG1 and PG2 strains, regarding the inorganic phosphate solubilization, the capacity to produce indolacetic acid (IAA) and ammonia (NH_3_), the utilization of 1-aminocyclopropane-1-carboxylic acid (ACC), and the possibility to grow in diverse stressed conditions such as osmotic and saline environment.

To test the capacity of bacteria to solubilize inorganic phosphate, bacterial cell suspension (5 μl, 1 × 10^8^ CFU mL^−1^) was spotted on plates with Pikovskaya agar medium with tri-calcium phosphate (Ca_3_(PO_4_)_2_), considering a triplicate for each plate. Following an incubation at 28 °C for 72 h, bacterial colonies with the clarification haloes was considere as positive for phosphate solubilization activity, and diameter of haloes were evaluated by Fiji software (ImageJ1.50i; Schneider et al. 2012).

Production of IAA was measured by using a colorimetric detection assay in liquid culture (Karnwal 2009). Isolates were cultured in Luria–Bertani (LB) broth, and then 300 μL of bacteria suspension (adjusted to 1 × 10^8^ CFU mL^−1^) were inoculated in 15 mL tubes filled with 3 mL of DF (Dworkin and Foster 1958) salt minimal broth with 150 μg mL^−1^ of L-Tryptophan. Tubes were maintained at 28 ± 2 °C on an orbital shaker (200 rpm, 48 h) in dark conditions. One mL of cell suspensions was centrifuged (4,000 rpm, 20 min, 4 °C) and 250 μL of supernatant were combined with 1 mL of Salkowski’s reagent (1.2% FeCl_3_ in 37% sulphuric acid). Samples were incubated twenty minutes at room temperature, using 96-well polystyrene dishes. A FluoStar Omega microplate reader was used to measure the absorbance of bacterial suspensions (Brescia et al. 2023). IAA concentrations were determined by the preparation of a standard curve using a range of 0.5–100 μg mL^−1^ of pure IAA, expressing the IAA amount in µg produced by bacterial suspensions at 0.5 McFarland scale density (1.5 × 10^8^ cells mL^−1^).

Evaluation of the utilization of ACC by bacterial strains (Li et al. 2011) was perforemd using 2 mL aliquots of LB cultures that were centrifuged at 8000 g for 5 min. The resulting pellets were then washed two times with 1 mL of DF medium (Dworkin and Foster 1958) and resuspended in 2 mL of DF medium with 3 mM ACC. Cultures were incubated at 28 °C, using an orbital shaker (200 rpm), for 24 h and 1 mL of each culture was then centrifuged at 8000 g for 5 min. One hundred µL of the supernatants (1:10 in DF medium) and 200 µL of each diluted supernatant, in addition to 200 µL of DF + ACC medium that was used as reference, were mixed to 400 µL of ninhydrin reagent and boiled for 30 min. Three aliquots of 180 µL for each strain as well as the reference samples were placed in the 96-well polystyrene plates, and the resulting Ruhemann’s Purple colour was evaluated by using a FluoStar Omega microplate reader (570 nm absorbance). A standard curve (in the range 0.005 and 0.05 mM) was used to assess ACC amount. In detail, the ACC-utilizing strains were grouped calculating the % of residual ACC compared with that of the DF-ACC medium without inoculation (Brescia et al. 2023). Production of ammonia (NH_3_) by the considered bacterial cultures were evaluated by using Nessler’s reagent (Cappuccino and Sherman 1999), following both the Abdelwahed et al. (2022) modified protocol and the original method. The two considered bacterial strains were grown in triplicate in peptone broth (10 mL) for 48 h in an incubator shaker at 30 ± 0.1 °C. After centrifugation (5 min, 6000 g), aliquots of 100 µL of each bacterial supernatant were mixed with 200 µL of Nessler’s reagent in 1.5 mL tubes and the mixture was incubated for 10 min, during which its colour turned to yellow to dark brown. After mixing, 33 µL aliquots of each reaction mixture were transferred to the wells in a 96-well plate and diluted with 198 µL of ultra pure water, and a FluoStar Omega microplate reader was utilized (450 nm absorbance) for estimating the extracellular production of NH_3_. The absorbance of a standard reference curve, prepared using ammonium sulphate ((NH _4_)_2_SO_4_) solutions (from 50 to 400 µM), was read along with samples. In the original method, greater amounts of bacterial supernatants (1 mL), Nessler’s reagent (9 mL) were utilized, keeping the same reaction timing and dilution ratio before transferring the samples to 1-mL cuvettes for absorbance measurements.

The two strains’ capacity to grow in osmotic and saline environments were also checked. The osmotic stress conditions were produced by using polyethylene glycol (PEG 6000) (Bandeppa et al. 2015). Bacterial strains were inoculated in liquid nutrient broth with glucose (NBG) medium containing 10%, 20%, 30%, 40% and 50% PEG 6000 (100 µl of a suspension at OD_600_ = 1) and put in a shaking incubator set at 28 °C and 120 rpm. NBG medium without PEG 6000 was used as a control.

To evaluate the growth in salt stress conditions, colonies were streaked on nutrient glucose agar (NGA) plates containing 2.5%, 5%, 7.5% or 10% NaCl (Nautiyal et al. 2000). NGA plates without any addition of NaCl (*i.e.*, 0.5% NaCl) were used as control. Samples were kept at 28 °C (in the dark), and bacterial growth was checked after 72 h. Number of replicates was three (n = 3) for each test. Putative pathogenicity of the two bacterial strain was also tested on tomato plants (Supplementary Information file).

### Biocontrol activity of bacterial strains against phytopathogens

Selected strains were checked for their capacity to inhibit the growth of *Fusarium oxysporum* f. sp. *lentis* and *Rhizoctonia solani* (provided by CNR-ISPA ITEM collection, Bari, Italy, and Dr. Alessandro Infantino, CREA-DC, Roma, Italy, respectively). Six-mm-diameter mycelial plugs obtained from 6-day-old cultures of the two fungal phytopathogens were put in the middle of a 9-cm-diameter Petri dish containing potato dextrose agar (PDA). After growing the bacterial strains in 2 mL of nutrient broth (NB) on an orbital shaker (200 rpm, 24 h, 28 °C), they (once that they have reached an optical density to OD_600_ = 1) were streaked (10µL) at the opposite sides (about 7 cm apart) of PDA plates containing the fungus. Plates containing the sole fungal isolate were used as controls. Each bacterial strain and each fungal control were cultured in triplicate for 6 days at 25 °C. The diameters of fungal colonies were evaluated to calculate the inhibition rate (% of 1 – dt/dc, where dt is the mean test fungal colony diameter and dc is the control one).

### Molecular characterization of bacterial strains

The DNA of the two considered strains (PG1 and PG2), grown overnight in NBG on a shaking incubator (28 °C, 180 rpm), was extracted using the E.Z.N.A.® Bacterial DNA Kit. Primers 27F (GAGAGTTTGATCCTGGCTCAG) (Melničáková et al. 2013) and 1495R (CTACGGCTACCTTGTTACGA) (Ventura et al. 2002) were used to amplify the 16S rRNA gene, using the protocol described in Supplementary Information file. The considered strains were also characterized for *nifH* encoding a nitrogenase as described in Supplementary Information file.

### Seed sterilization and plant growth conditions

Seeds of two different pea genotypes commonly cultivated by Tunisian farmers were used: *cv*. Merveille de Kelvedon a medium-early, sweet and highly sought-after variety easy to grow, and *cv.* Lincoln, which is a medium-early variety, excellent for home gardens and organic production and it is adapted to the edapho-climatic conditions of Tunisia. Seeds were sterilized in 70% v/v EtOH for 60 s and in 2.5% v/v sodium hypochlorite (30 min), washed five times with sterilized water, put in Petri dishes on moist sterilized filter paper, and stored for 7 days at 25 °C (in the dark). Thirty Lincoln seedlings were transplanted to pots (0.7 L) containing sterilized quartz sand, while 30 Kelvedon and 60 Meraviglia d’Italia seedlings were transplanted to pots (1.4 L) containing sterilized quartz sand and coconut fiber (1:1 v/v). Plants were watered to compensate for evapotranspiration for one month, then twice *per* week with water and once a week with 50 mL ½ strength Hoagland solution (Hoagland and Arnon 1950), and they were grown in a greenhouse following the natural sun photoperiod (September–November 2022).

### Bacterial inoculation in pea plants

After 28, 13 and 10 days after transplanting for Merveille de Kelvedon, Lincoln and Meraviglia d’Italia, respectively, plants were inoculated with the strains PG1 and PG2. Inoculum was prepared growing PG1 and PG2 for two days in NBG using a shaking incubator set at 26 °C (180 rpm). When PG1 and PG2 cultures reached the OD_600_ of 3.5, equivalent to approximately 1.9 × 10^9^ and 1.2 × 10^9^ CFU mL^−1^, plants were inoculated by pipetting 1 mL of culture in each pot at 1–2 cm depth, with pipette tips maintained at 45° to reach the root apparatus. Ten independent plants (respresenting the biological replicates) were prepared for each bacterial strain, in addition to other ten uninoculated control plants for genotypes Merveille de Kelvedon and Lincoln. Conversely, twenty independent biological replicates were used for each bacterial strain for Meraviglia d’Italia, in addition to twenty uninoculated control plants.

### Salt application

After 32, 32, 23 days since transplanting, for Merveille de Kelvedon, Lincoln and Meraviglia d’Italia, respectively, 50 mL of NaCl (50 mM) starting solution was dispensed in each pot (n = 5 for Merveille de Kelvedon and Lincoln, n = 10 for Meraviglia d’Italia). Plants were subjected to salt stress watering them two times a week starting with a solution containing 100 mM NaCl and enhancing this concentration of 50 mM every three days to avoid salt shock, until reaching 200 mM NaCl as a stress condition (Pollastri et al. 2018). After 15 days from transplanting (*i.e.,* 47, 47 and 38 days after transplanting for Merveille de Kelvedon, Lincoln and Meraviglia d’Italia, respectively) the experiment was stopped.

### Biometrical and physiological measurements of plants

Before the beginning of the stress treatments, as well as during the stress period, biometric and physiological measurements were carried out. Height, node number, chlorophyll content by SPAD (Opti-Sciences) and stomatal conductance (*g*_s_) with LI-COR 600 (Ecosearch) were measured. At harvest (*i.e.,* at the end of the stress treatment, when plants showed clear stress symptoms such as chlorosis), shoots were collected in liquid nitrogen and kept at − 80 °C for gene expression analysis and metabolic stress markers, while roots were oven-dried dried at 70 °C for 48 h for biomass determination. For Meraviglia d’Italia plants, in addition to those analyses, shoot biomass was also determined using the additional set of prepared replicates (n = 5). Pod number was recorded for all genotypes.

### Biochemical analysis of *P. sativum* stress markers

Lipid peroxidation was evaluated through the malondialdehyde (MDA) content, resulting from the thiobarbituric acid (TBA) reaction (Heath and Packer 1968). Hydrogen peroxide (H_2_O_2_) amount was determined using the KI method (Loreto and Velikova 2001), while free proline levels were quantified using the ninhydrin reaction (Bates et al. 1973). Details have been reported in the Supplementary Information file.

### Quantitative gene expression analysis in *P. sativum* leaves

Three biological replicates for each stress treatment (non-stressed, NS; salt stress, S) and for each inoculum (PG1, PG2, not inoculated control) were considered for the evaluation of gene expression. Total RNA was extracted from leaves (100 mg) of each biological replicate considered for gene expression (n = 3) utilizing the Spectrum Plant Total RNA extraction kit (Sigma-Aldrich) with slight modifications. RNA was quantified by NanoDrop 1000 spectrophotometer and then was treated with TURBO™ DNase kit following the routine DNase treatment (Thermo Fisher Scientific). DNA contamination absence was verified prior to proceed with the cDNA synthesis, by using pea *PsPP2A* specific primers in one-step RT-PCR (Knopkiewicz and Wojtaszek 2018) (Table 1). Total RNA was used to generate the cDNA, according to the SuperScript II Reverse Transcriptase® (Invitrogen) protocol using random primers. Each RT-qPCR reaction was conducted on a total volume of 10 μL, containing 1 μL diluted cDNA (dilution 1:2), 5 μL Power SYBR Green PCR Master Mix; Biorad), 3.6 μL of water and 0.2 μL of each primer (10 μM), using a 96-well plate. Primers are reported in Table 1. Reactions were performed using the Connect™ Real-Time PCR Detection System (Bio-Rad Laboratories). Thermal cycling conditions were as follows: 95 °C for 10 min, followed by 40 cycles at 95 °C for 15 s and 60 °C for 1 min. Pea target transcripts in leaves were quantified after normalization with two reference genes: *PsPP2A* encoding a protein phosphatase 2A (Knopkiewicz and Wojtaszek 2018) and *PsEF1α* coding for an elongation factor gene (NM_001427569.1). Three independent biological replicates were used (with the exception of control of salt stressed plants of genotype Meraviglia d’Italia for which two biological replicates were considered) as well as two technical replicates for each biological one. The candidate gene expression was calculated using the equation 2^−ΔΔCT^ (Livak et al. 2001).

**Table 1:** List of the primers used in the study and the putative function of the targeted genes

Putative function	Gene name	Forward primer	Reverse Primer	References
Elongation factor 1 alpha	*PsEFα1*	AGACCCTCGGACAAGCCCCT	ACCGGGCTTCACAACACCAGT	This work
protein phosphatase 2A	*PsPP2A*	CGGCTGCTGCGTGATAATG	TGGTAGAATATGCTGAATGGCTAGTT	Knopkiewicz and Wojtaszek (2018)
Superoxide dismutase	*PsSOD*	CCATCATAGGAAGGGCTGTTGT	CGTGACCACCTTTCCCAAGA	Gupta et al. (2021a)
Catalase	*PsCAT*	TCACAGAGGCACAGACTTGG	GACCTCCTCATCCCTGTGAA	Gupta et al. (2021a)
Ascorbate Peroxidase	*PsAPX1*	GAGATTACCGGTGGACCTGA	CTAAGCCCCATAGCCTTTCC	Gupta et al. (2021a)
Chlorophyll a/b binding protein	*PsCHL A/B*	CGGACCAGACCGTGTTAAGT	TGGATGACTTCAAGCTCACG	Gupta et al. (2021a)

### Statistical analysis

R software (version 4.1.1) was used for statistical analysis. Effects of pea genotype, applied stress, and inocula, on biochemical, biometric and physiological data at the end of the experiment were tested with analyses of variance (three-way ANOVA). Differences among treatments within the plant genotypes were statistically assessed using one-way ANOVA and a Tukey’s HSD test, transforming data when necessary to fulfill ANOVA assumptions. A probability level of *p*-value < 0.05 was considered for all tests. Principal component analysis (PCA) was utilized for comparing both the biometric and physiological data, as well as the biochemical ones. R software (version 4.1.1) was utilized to calculate the Pearson correlation matrix. The Relative Expression Software Tool REST© 2009 v. 2.0.13 (Qiagen) (Pfaffl et al. 2002) was used for performing statistical analysis on gene expression data, using 0.05 as significance of *p*-value. To conduct the correlation analysis (Pearson correlation) between biochemical stress marker and gene expression (relative expression, ΔCT) data, a square root transformation was carried out to ensure normality and improve the linearity assumption. This analysis was conducted using the R package "PerformanceAnalytics". Specifically, the "chart.Correlation" function of this package was used. To assess the significance of the observed correlations, *p*-values were calculated for each correlation coefficient, setting it at 0.05. The *p*-values were obtained using the built-in functionality of the "chart.Correlation" function.

## Results

### Characterization of bacterial strains

**Fig. 1:**
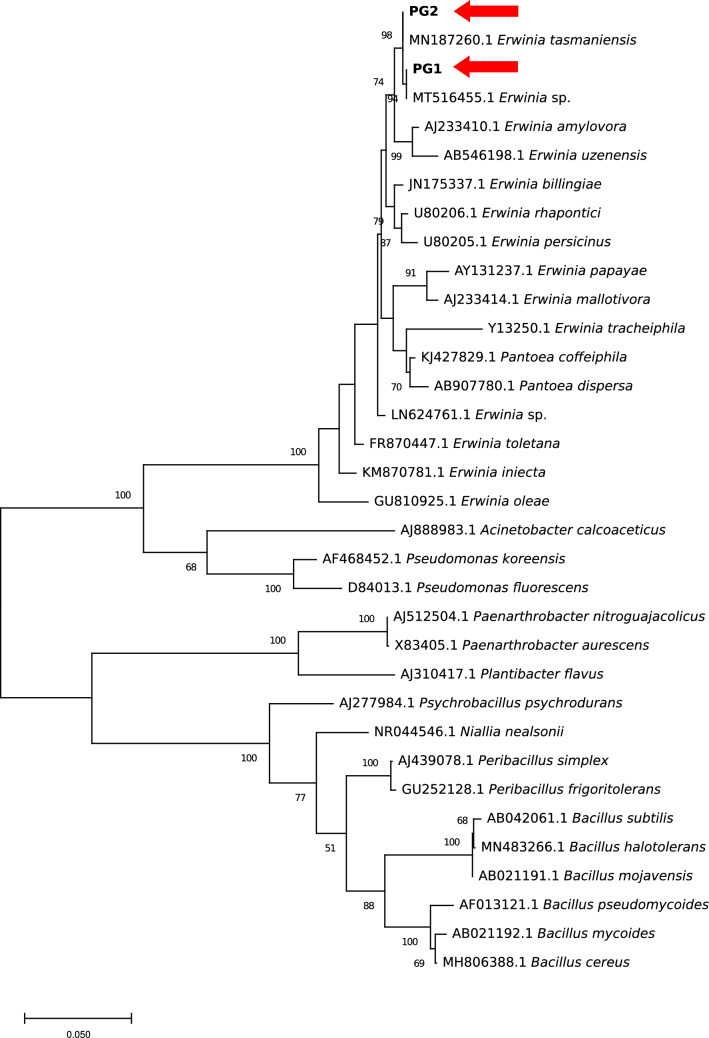
Maximum parsimony bootstrap consensus tree obtained from 16S rRNA gene sequences. The numbers indicate bootstrap values and are given only for > 50%

Biocontrol activity showed that six-days-old co-cultures of the phytopathogenic fungus *F. oxysporum* f. sp. *lentis* with isolates PG1 and PG2 showed on average 26.4 ± 0.7% reduction in fungal growth compared with controls (represented by the sole fungal colonies), with no significant differences among bacterial isolates. On the other hand, no growth reduction was recorded when *R. solani* was grown in the presence of PG1 or PG2. Molecular and biochemical characterization of the two selected strains was carried out by the 16S rRNA gene sequencing and evaluation of PGP traits. The sequencing of the PCR amplicons resulted in two sequences of 1368 bp (PG1) and 1388 bp (PG2) in length. These sequences were submitted in the GenBank database (accession number OQ919144 and accession number OQ919145 for PG1 and PG2, respectively) and compared against the NCBI database (16 March 2023). The two strains showed the highest identity with *Erwinia tasmaniensis*, followed by other species of *Erwinia* (Table 2). The phylogenetic tree confirmed the BLAST outputs, clustering the two strains close to *E. tasmaniensis* (Fig. 1). Based on PCR performed with primers for *nifH*, PG2 produced an amplicon between 300 and 400 bp, while PG1 did not. The amplicon was sequenced, showing 98.78% of identity with *Rhizobium* sp. KNb1 *nifH* (e-value of 2e^−119^) and submitted to NCBI GenBank (accession number OR083229). Tests in pure culture showed that both bacterial strains had the capacity to produce IAA and ACC deaminase activity and that PG1 showed the capacity to solubilize phosphate (Table 3). The ability of producing extracellular NH_3_ by both bacterial strains could be visually assessed by observing the development of the orange colour by surnatant-based reactions mixtures after the addition of Nessler’s reagent. An apparent darker colour, suggesting higher NH_3_ production, was detectable in the reaction mixtures obtained from the strain PG2. Unfortunately, the absorbance data recorded from both the original method and from the 96-well plates assays of supernatants from the two bacterial cultures were very variable among replicates, even when higher dilution levels (1:8 and 1:10, in place of 1:5) were used, and showed no significant differences among strains, whose extracellular NH_3_ production ranged between 200 and 270 µM (not shown). In the salt stress test, PG1 was able to grow until a NaCl concentration of 7.5%, while PG2 could tolerate 10% NaCl, despite showing a reduced growth. Additionally, PG1 was able to grow on 40% PEG plates, while PG2 tolerated up to 30% PEG (Table 3).

**Table 2:** Best 5 hits based on PG1 and PG2 16S rRNA gene sequences obtained with primers 27F-1495R and compared against NCBI database RNA_typestrains/16S_ribosomal_RNA

	Blast result	Coverage (%)	*e*-value	% identity	Accession number
PG1	*Erwinia tasmaniensis* Et1/99	100	0.0	99.12	NR_074869.1
	*Erwinia tasmaniensis* Et1/99	100	0.0	99.05	NR_042422.1
	*Erwinia endophytica* strain BSTT30	100	0.0	98.61	NR_148650.1
	*Erwinia billingiae* strain Billing E63	100	0.0	98.47	NR_104932.1
	*Erwinia billingiae* strain LMG 2613	100	0.0	98.49	NR_118431.1
PG2	*Erwinia tasmaniensis* Et1/99	100	0.0	99.14	NR_074869.1
	*Erwinia tasmaniensis* Et1/99	100	0.0	99.06	NR_042422.1
	*Erwinia endophytica* strain BSTT30	100	0.0	98.63	NR_148650.1
	*Erwinia billingiae* strain Billing E63	100	0.0	98.49	NR_104932.1
	*Erwinia billingiae* strain LMG 2613	100	0.0	98.42	NR_118431.1

**Table 3:** Results obtained by biochemical and physiological tests on bacterial strains PG1 and PG2 isolated from root nodules

Strain	IAA production (µg)	P solubilization halo (mm)	Residual ACC (%)	Fungal growth reduction activity in co-culture (% of control)		Bacterial growth at different NaCl concentrations (%)					Bacterial growth at different PEG6000 concentrations (%)					
				*F. oxysporum*	*R. solani*	0.5	2.5	5.0	7.5	10.0	0	10	20	30	40	50
PG1	3.55 ± 0.07	31.2 ± 0.6	9.2 ± 0.6	25.7 ± 0.5	ND	+	+	+	+	−	+	+	+	+	+	−
PG2	4.08 ± 0.06	< 5	12.1 ± 0.5	27.1 ± 0.4	ND	+	+	+	+	±	+	+	+	+	−	−

Data represent the average of three replicates ± SE. The growth on different concentrations of salt and PEG6000 are indicate with symbol + and −, where + indicates growth, – the absence of growth and ± stunted growth

### *P. sativum* biometric and physiological parameter evaluation

**Fig. 2:**
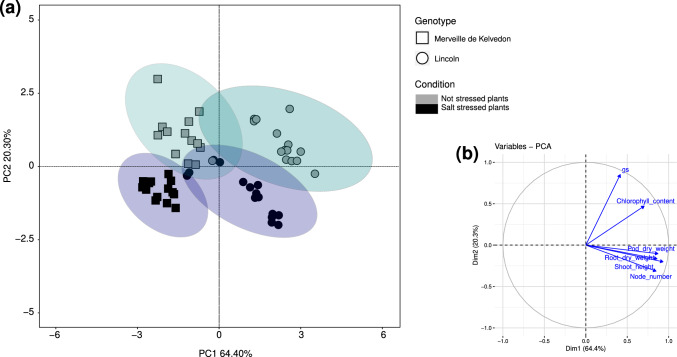
Multivariate statistical analysis of physiological (chlorophyll content and *g*_s_) and biometric (shoot height, node number, root and pod dry weight) data on Merveille de Kelvedon and Lincoln (n = 5). In (**a**), Principal component analysis (PCA) of samples; in (**b**), projection of variables, where angles are interpreted as correlations. The angle between two variable vectors represents the degree of correlation between them: adjacent (angle less than 90°) showed highly correlated variables, angle more than 90° showed uncorrelated ones

**Fig. 3:**
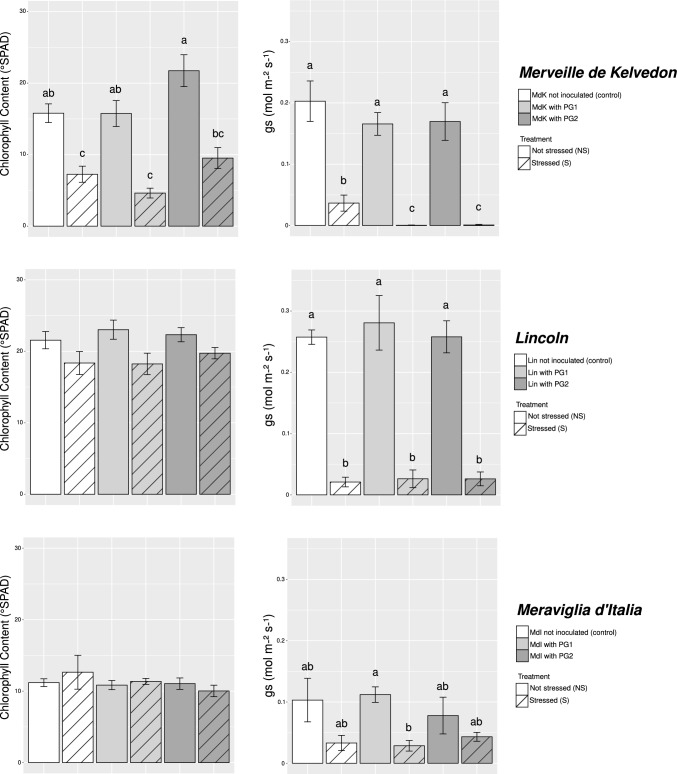
Physiological data (chlorophyll content °SPAD and *g*_s_ mol m^−2^ s^−1^) in Merveille de Kelvedon (n = 5), Lincoln (n = 5) and Meraviglia d’Italia (n = 10). All data are expressed as mean ± SE (standard error). Letters are plotted on the base of Tukey’s test; different ones mean significant differences (*p*-value < 0.05). The absence of letters means absence of significant differences

Biometric and physiological measures were taken during plant growth period, namely shoot height, node number and chlorophyll content, together with stomatal conductance, *g*_s_. Then, after harvest the pod number and pod, root and, only for Meraviglia d’Italia, shoot dry weights were checked. The analysis of variance, considering genotype, inocula and stress, showed significant differences depending on the genotype for all the considered biometric and physiological parameters, and on the applied stress for height and physiological parameters (Table S1). The PCA using biometric and eco-physiological data, recorded at the end of the experiment, suggests that three genotypes performed in a variable manner (Figure S1). Considering Merveille de Kelvedon and Lincoln, genotypes employed in Tunisia, the PCA showed a differential response to salt (Fig. 2). A significant positive correlation was found between shoot height and node number, pod dry weight and root dry weight, shoot height and root dry weight, shoot height and pod dry weight, and node number and pod dry weight (Table S2). When data from each genotype were analyzed independently, no significant differences were detected among the treatments for all the considered biometric parameters (Table S3). Concerning the physiological parameters, in Merveille de Kelvedon the chlorophyll content was significantly lower in salt stressed uninoculated and PG1-inoculated plants, compared with their non-stressed controls, while in the other two genotypes there were no statistical differences (Fig. 3). Pea plants belonging to Merveille de Kelvedon, grown without salt stress and treated with the strain PG2, had the tendency to show higher chlorophyll contents than those inoculated with PG1 (Fig. 3).

Relating to *g*_s_ values, in Merveille de Kelvedon and Lincoln the salt stressed plants showed significantly lower values than their controls, and a severe reduction of this variable under stress, irrespective of bacterial inoculation. Conversely, stomatal conductance (*g*_s_) data obtained from Meraviglia d’Italia genotype were not significantly diverse between not stressed and stressed uninoculated and PG2-inoculated plants, while PG1 bacterial inoculation led a significant difference between not stressed and stressed plants (Fig. 3).

### Biochemical analysis of *P. sativum* stress markers

**Fig. 4:**
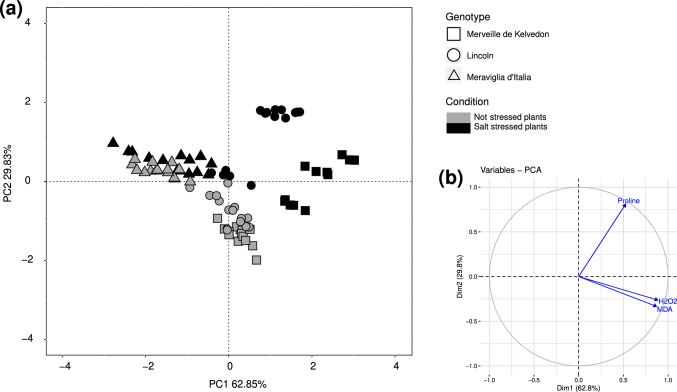
Multivariate statistical analysis of biochemical data (MDA, H_2_O_2_, proline content) on the three genotypes (Merveille de Kelvedon, Lincoln and Meraviglia d’Italia). In (**a**), Principal component analysis (PCA) of samples; in (**b**), projection of variables, where angles are interpreted as correlations. The angle between two variable vectors represents the degree of correlation between them: adjacent (angle less than 90°) showed highly correlated variables, angle more than 90° showed uncorrelated ones

The three-way ANOVA showed significant differences for each considered factor (genotype, applied stress, inoculation) (Table S4). The PCA carried out using biochemical parameters highlighted the differential responses of the three pea genotypes: while in Merveille de Kelvedon and Lincoln plants there was a separation between not stressed and stressed plants, in Meraviglia d’Italia this separation was less evident (Fig. 4). Considering each genotype separately, salt stress affected MDA and proline quantity in Merveille de Kelvedon, significantly increasing their values, while in Lincoln and Meraviglia d’Italia only slight differences were detected between stressed and not stressed plants (Table 4). Considering bacterial inoculation, salt stressed Merveille de Kelvedon plants inoculated with PG1 consistently showed the highest values of MDA, H_2_O_2_ and proline, while in the presence of PG2 only H_2_O_2_ content was significantly enhanced compared with both uninoculated stressed plants and not stressed ones (Table 4). Lincoln plants showed a largely different behavior. Significantly higher values of MDA were detected in not stressed PG1-inoculated plants and in stressed plants treated with PG2, compared with stressed uninoculated plants that showed the lowest value. H_2_O_2_ content was similarly low in stressed plants treated with PG2 and in not stressed uninoculated plants, while the highest value was found in stressed plants inoculated with PG1. Relating to proline, the highest value was found in stressed plants inoculated with PG1 and the lowest one in not stressed plants inoculated with PG2 (Table 4). In Meraviglia d’Italia, the lowest MDA value was recorded in stressed plants treated with PG2. Conversely, significantly higher values were detected in the other stressed plants, irrespective of inoculation, and in not stressed controls. No significant differences were found among treatments for H_2_O_2_ content, while the highest proline values were detected in stressed plants inoculated with PG1, which were significantly different from those found for all the other treatments (Table 4).

**Table 4:** Biochemical parameters (malondialdehyde, MDA; hydrogen peroxide, H_2_O_2_; proline) measured in three different pea genotypes inoculated or not with bacterial strains under salt stress (S) or control not stressed (NS) conditions

Genotype	Growth condition	Bacterial inoculation	MDA (nmol g^−1^ FW)	H_2_O_2_ (μmol g^−1^)	Proline (μmol g^−1^)
Merveille de Kelvedon	NS	PG1	16.75 ± 1.28 c	0.06 ± 0.01 cd	7.78 ± 1.15 e
		PG2	16.48 ± 1.44 c	0.06 ± 0.01 cd	10.06 ± 0.88 de
		C	16.27 ± 1.26 c	0.05 ± 0.01 d	10.91 ± 0.93 d
	S	PG1	26.57 ± 1.39 a	0.09 ± 0 ab	133.5 ± 1.71 a
		PG2	19.97 ± 1.35 b	0.09 ± 0.01 a	72.18 ± 1.04 b
		C	20.55 ± 1.16 b	0.07 ± 0.01 bc	29.17 ± 1.47 c
Lincoln	NS	PG1	15.69 ± 0.74 a	0.05 ± 0 bc	15.58 ± 1.32 e
		PG2	14.59 ± 1.35 ab	0.06 ± 0.01 ab	10.78 ± 1.35 f
		C	13.77 ± 1.37 ab	0.04 ± 0 cd	18.93 ± 1.23 d
	S	PG1	14.32 ± 0.54 ab	0.07 ± 0.01 a	212.88 ± 1.14 a
		PG2	15.48 ± 1.05 a	0.04 ± 0.01 d	35.39 ± 1.89 c
		C	13.13 ± 0.7 b	0.06 ± 0.01 ab	171.46 ± 1.37 b
Meraviglia d’Italia	NS	PG1	9.12 ± 0.64 b	0.03 ± 0	20.61 ± 1.28 d
		PG2	8.58 ± 0.82 b	0.03 ± 0.01	21.63 ± 1.76 cd
		C	10.98 ± 0.83 a	0.03 ± 0.01	21.36 ± 0.78 cd
	S	PG1	12.15 ± 1.07 a	0.03 ± 0.01	38.95 ± 1.41 a
		PG2	6.71 ± 1 c	0.03 ± 0.01	23.67 ± 1.54 c
		C	11.51 ± 0.63 a	0.03 ± 0	26.66 ± 1.55 b

All data are expressed as mean ± SD (standard deviation). Different letters indicate significant differences (*p*-value < 0.05) within genotype. In Merveille de Kelvedon n = 5 in CS, CNS and PG2NS, in the other treatments n = 4; in Lincoln n = 5 with the exception of the proline analysis were n = 4 in CNS; in Meraviglia d’Italia n = 5, with the exception of PG1S where n = 4 and in proline CNS n = 4

### RT-qPCR results

**Fig. 5:**
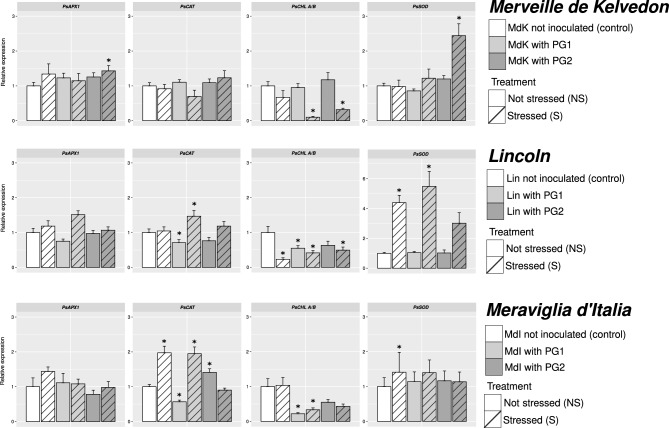
Relative expression levels (Fold change ± SE), calculated following the equation 2^−ΔΔCT^ of the four considered genes in Merveille de Kelvedon, Lincoln and Meraviglia d’Italia (n = 3, with the exception of stressed control in Meraviglia d’Italia where n = 2). Asterisks mean significant differences in comparison to control (non-stressed and uninoculated) plants, according to Pair Wise Fixed Reallocation Randomization Test © performed by REST© 2009

To verify the plant response in the presence of salt, the expression of four genes putatively regulated by salt stress (Gupta et al. 2021a) was evaluated. The four genes include a gene coding for a superoxide dismutase (*PsSOD*), a catalase *(PsCAT*), an ascorbate peroxidase (*PsAPX1*) and a chlorophyll a/b binding protein (*PsCHL A/B*). Relative expression of *PsAPX1* did not show differences among salt and/or bacterial treatments and control plants in all the genotypes, with the exception of Merveille de Kelvedon, where it was significantly up-regulated in stressed PG2-inoculated plants (Fig. 5). The transcription of *PsCAT* did not show differences among treatments in plants belonging to Merveille de Kelvedon genotype, while it was down-regulated in PG1-treated not stressed plants of Lincoln genotype, and up-regulated in stressed plants inoculated using the same isolate. In Meraviglia d’Italia genotype, this gene was up-regulated in salt stressed controls (*p*-value 0.007), stressed PG1-treated plants and in PG2-inoculated plants, while it was down-regulated in PG1-inoculated plants (Fig. 5).

The gene *PsCHL A/B* was down-regulated in all the genotypes, although differently modulated by treatments. Significant down-regulation was detected in stressed Merveille de Kelvedon plants inoculated with PG1 and PG2, in all treatments of Lincoln genotype with the exception of non-stressed PG2-inoculated plants, and in PG1 treatments of both not stressed and stressed plants in Meraviglia d’Italia (Fig. 5). In Merveille de Kelvedon genotype, the *PsSOD* encoding gene was up-regulated only in stressed PG2-inoculated plants, while significant up-regulation in stressed controls and in PG1-inoculated and control stressed plants was detected in plants belonging to Lincoln and Meraviglia d’Italia genotypes, respectively (Fig. 5).

### Gene expression and biochemical data correlation

**Fig. 6:**
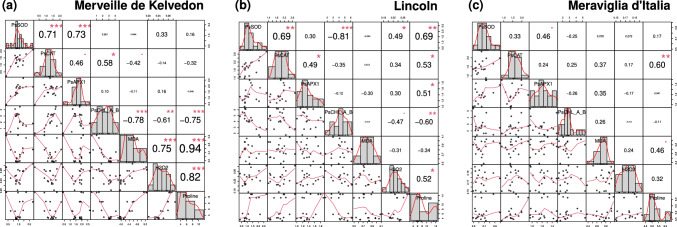
Correlation matrices of biochemical and target gene expression data in the three genotypes. Matrix of data in Merveille de Kelvedon (**a**), Lincoln (**b**) and Meraviglia d’Italia (**c**). Each diagonal subplot shows the distribution of data of the considered variable as a grey histogram. Scatterplots of each pair of variables with a least-squares reference line (red) are also reported. The slope of red line corresponds to the Pearson correlation coefficient. Numbers in the matrix represent the Pearson correlation coefficients (r). Font size used for correlation coefficients mirrors the degree of correlation. Red asterisks showed the *p*-value of the correlation ( = *p* < 0.1, * = *p* < 0.05, ** = *p* < 0.01, *** = *p* < 0.001)

Data obtained by gene expression and biochemical analyses were correlated, putting in evidence that in Merveille de Kelvedon and Lincoln nine correlations were detected, while in Meraviglia d’Italia only one. In detail, the significant positive correlations in Merveille de Kelvedon were between the relative expression of *PsSOD* and *PsCAT* and of *PsSOD* and *PsAPX1* and among the three stress markers (MDA, H_2_O_2_ and proline), while the relative expression of *PsCHL A/B* was significantly negatively correlated with the same stress markers. The significant positive correlations in Lincoln were between the relative expression of *PsSOD* and *PsCAT* and of *PsCAT* and *PsAPX1*, these three genes with proline, *PsSOD* with H_2_O_2_, H_2_O_2_ with proline, while the negative correlations were between the relative expression of *PsSOD* and *PsCHL A/B* and of *PsCHL A/B* with proline (Fig. 6). The only positive correlation in Meraviglia d’Italia was between the relative expression of *PsCAT* and proline.

## Discussion

Plants can activate different mechanisms to protect themselves from stressful conditions, such as in high salinity (Raza et al. 2019). It has been widely reported that the application of beneficial bacteria can increase salt tolerance in different crops such as tomato, chickpea, French bean, canola, wheat, rice, maize, potato, and pepper (Gupta et al. 2022; Orozco-Mosqueda et al. 2020). Gupta et al. (2021b) recently demonstrated in a pot experiment that *Bacillus subtilis* RhStr_71, *Bacillus safensis* RhStr_223, and *Bacillus cereus* RhStr_JH5 improved pea plant growth, increasing the abundance of osmoprotectant and antioxidant compounds and reducing oxidative stress level due to salt. In addition, combination of PGPB and mycorrhizal fungi has been documented to mitigate the negative salt effects, enhancing *Lathyrus cicero* plant growth (Gritli et al. 2022).

Here, two bacteria isolated from Tunisian pea root nodules have been characterized molecularly and biochemically, and the effects of these strains on three pea genotypes in both optimal and salt stress conditions have been evaluated.

### Characterization of bacterial strains

Sequencing of 16S rRNA gene allowed to verify that the two bacterial isolates both resulted to belong to *Erwinia* sp., phylogenetically close to *Erwinia tasmaniensis.* This species was first isolated from flowers and bark of apple and pear trees in Australia, showing that bacteria belonging to this species were non-phytopathogenic (Geider et al. 2006). The tests carried out by challenging tomato plants with the two isolates showed the absence of disease symptoms, confirming the non-pathogenicity of these bacterial strains, at least on two plant species (pea and tomato). The presence of non-pathogenic *Erwinia* species, among which *E. tasmaniensis* strains from different countries, was already reported (Sagar et al. 2018). Available genome sequences support these data, suggesting that non-pathogenic isolates lack the set of genetic factors needed for tissue invasion (Kube et al. 2010). Although some genetic traits involved in the induction of hypersensitive responses (HR) was still found in *E. tasmaniensis* strain Et1/99, able to start HR on tobacco leaves, no virulence on host apple and pear was detected (Kube et al. 2010; Palacio-Bielsa et al. 2012). The capacity of the two *E. tasmaniensis* strains to control the growth of a legume-derived isolate of *F. oxysporum* was detected in vitro, suggesting that screenings for antagonistic activity of nodule-associated microorganisms against microbial pathogens should be carried out, both in vitro and in vivo, to explore such potentially useful traits.

The molecular characterization of the bacterial strains was based on the search for the *nifH* gene (coding for nitrogenase), which is the most used marker gene for the identification nitrogen-fixing bacteria (Gaby and Buckley 2012). Here, its presence was detected in PG2, suggesting the potential capability of this strain to reduce nitrogen into ammonia.

On the basis of the phenotypic characterization, PG1 may represent a strain with an applicative potential due to its capacity for solubilysing phosphate, producing IAA and ACC deaminase, and to its ability to grow in presence of NaCl or PEG. The presence of these traits is common to other interesting PGPB such as *Planomicrobium* sp. MSSA-10 (Shahid et al. 2018), able to release organic acids in the rhizosphere resulting in increased P availability to plant roots, to regulate plant ethylene level and to produce root growth-promoting hormone. The observed features were also reported for another strain of *Erwinia* sp. (KP226572) that demonstrated to enhance wheat growth (Sagar et al. 2018). The bacteria producing ACC deaminase can help plant development, especially upon environmental stressful conditions (Ali et al. 2014). It was demonstrated that plants had higher levels of ethylene due to higher levels of ACC: in the presence of PGPB showing ACC deaminase activity, the ethylene levels decreased, leading to the production of α-ketobutyrate and ammonia (Gupta et al. 2022; Orozco-Mosqueda et al. 2020).

### Effects of salt stress on biometric and physiological parameters of *P. sativum* plants

Although each genotype showed a different behavior, neither salt stress nor the presence of bacterial inoculation led to significant effects on growth parameters and root biomass. Previous studies showed that pea growth resulted negatively affected by salt treatments exceeding 50 mM and that variable responses could be detected among genotypes, depending on their tolerance (Shahid et al. 2011, 2012). A positive correlation between pod dry weight and root dry weight, and between pod dry weight and shoot height, was observed in Merveille de Kelvedon and Lincoln plants, suggesting that the tendency for more developed root and foliar systems can be associated with heavier pods. This result is in line with the fact that well-developed roots are generally expected to support nutrition, growth and development of the whole plant, as previously observed in other legume species (Gopalakrishnan et al. 2016).

Salt stress dramatically decreased the *g*_s_ values in Merveille de Kelvedon and Lincoln pea genotypes, independently on the inoculation, in agreement with studies showing that reductions in stomatal conductance, along with photosynthetic rates, are common plant response to salt stress, being these variables affected by Na^+^/K^+^ ratio and turgor decline (Hernández et al. 2000; Irshad et al. 2021). A less severe *g*_s_ variation was observed in Meraviglia d’Italia plants, suggesting that this genotype has a different stomatal behaviour with respect to the Tunisian *cv*. (Hochberg et al. 2018).

### Stress marker evaluation

Three already known markers for stress, *i.e.,* MDA, H_2_O_2_, and proline, were also evaluated. Large increases in MDA under salt stress were consistently detected in the Merveille de Kelvedon, particularly in the presence of the PG1 isolate; the same isolate also induced enhanced MDA levels in Meraviglia d’Italia stressed plants, whose uninoculated controls were unaffected by salt stress. Increase of MDA under salt stress was previously reported for different legume species, *i.e.*, pea, bean and lentil (Noreen and Ashraf 2009; Taïbi et al. 2016; Yasir et al. 2021). High concentration of MDA mirrors membrane lipid peroxidation, but it can also be correlated to an acclimation process signal, able to activate regulatory correlated to plant defense and growth, and to protect against oxidative stresses (Morales and Munné-Bosch 2019). Conversely, low concentrations of MDA have been related to the less active lipid peroxidation in salt-tolerance mechanisms. MDA levels were significantly reduced by inoculation with the isolate PG2 in stressed Meraviglia d’Italia plants, in the absence of changes in H_2_O_2_ content. As MDA may play different, and largely unexplored, physiological roles, further studies are needed to reveal the impact of root-associated microorganisms on its accumulation. An increase of H_2_O_2_ concentration was detected in plants belonging to genotypes Merveille de Kelvedon and Lincoln under salt stress, according to studies already present in literature (Hernández et al. 2000; Irshad et al. 2021). Inoculation of salt stressed pea plants of both genotypes with PG1 increased H_2_O_2_ values, compared with uninoculated controls, while strain PG2 showed opposite effects on H_2_O_2_ levels, which were increased or decreased in salt stressed Merveille de Kelvedon or Lincoln pea plants, respectively. Thus, in Lincoln genotype the presence of PG2 bacterial strain might have reduced the plant stress status. In this regard, Neshat et al. (2022) suggested that the application of PGPR markedly decreased H_2_O_2_ levels, particularly under 100 mM salinity stress in canola plants. This reduction may be linked to the inhibition of Na^+^ uptake through roots and a concurrent decrease in K^+^ levels. Differential responses among genotypes and the lack of salt stress-related variations in H_2_O_2_ concentrations were previously reported for other pea cultivars (Noreen and Ashraf 2009). It is worth noting that, at high level, H_2_O_2_ is known to provoke oxidative damages to biomolecules, causing cell death, but at lower concentration it may work as a signaling molecule (Černý et al. 2018). The accumulation of such molecules in response to stress factors and microbial inoculants has proven highly dependent on plant and microbial identity and on their specific ability to modulate the multiple factors involved in stress response. Additionally, the regulation of this and other ROS depends on their generation and degradation, and on their neutralization rates by plant antioxidants. Results obtained on uninoculated plants of all pea genotypes confirmed that proline increases in the presence of osmotic stress. This amino acid plays an osmoprotective function, together with chaperone and antioxidant signal regulating function, whereas its specific role in response to stress in plants is still under debate (Szabados and Savoure 2010; Spormann et al. 2023). It is worth noting that Meraviglia d’Italia plants showed higher values of proline in not stressed conditions compared to the other two genotypes, and only a slight increase in proline content under stress. This result, together with the low MDA content detected in both not stressed and stressed Meraviglia d’Italia plants, may suggest a different response to salt stress for this genotype that harbors an enhanced basal level of proline compared to Merveille de Kelvedon and Lincoln. Additionally, *g*_s_ data of this genotype showed lower values in not stressed conditions compared to the other two genotypes. This reduced stomatal conductance could suggest a preventive and protective strategy against water loss by Meraviglia d’Italia plants. A recent work on the combined effect of drought and salinity on forage pea plants demonstrated that tolerant pea genotypes harbored high concentration of proline (Demirkol and Yilmaz 2023). Other studies reported that the concentration of osmolytes and antioxidant enzymes in pea play an important role in salt tolerance potential (Farooq et al. 2017; Shahid et al. 2022). It is worth noting that the inoculation with the strain PG1 in Merveille de Kelvedon and Lincoln plant genotypes induced the largest accumulation of proline in response to salt stress (17- and 14-fold, respectively, over the not stressed Merveille de Kelvedon- and Lincoln-inoculated plants). It has been documented that when salt stress mitigation measures are used, including the use of PGPB, proline levels often rise (Spormann et al. 2023). However, it is still under debate if proline accumulation results from changed redox balance and hormone metabolism or if it is a component of the tolerance-inducing process (Spormann et al. 2023). In our experiment, PG2 was less able to enhance proline accumulation under stress, as only a slighter increase in stressed plants was observed compared to inoculation with PG1, suggesting that PG2 inoculated plants may not need high proline accumulation. An explanation could be that these plants have an increase activity in proline degradation or that other osmotic solutes are involved in the response to stress (Szabados and Savouré 2010).

### Gene expression and correlation analysis

To cope with the oxidative damage caused by salt, plants have developed defenses based on antioxidant enzymes such as SOD, POX, CAT (Hasanuzzaman et al. 2020). Here, expression levels of these genes were strictly dependent on the considered plant genotype-bacterial strain combination. In previous works, *P. sativum* plants inoculated with different isolates belonging to *Bacillus* or *Pseudomonas* showed an up-regulation of the genes coding for several antioxidant enzymes in comparison to uninoculated salt-stressed plants (Gupta et al. 2021a; Sofy et al. 2021). Here, inconsistent up-regulation of *PsCAT* and *PsSOD* was detected under salt stress/bacterial treatments in genotypes Lincoln and Meraviglia d’Italia, while *PsCHL A/B* was down-regulated or not significant regulated, suggesting a diverse response to salt stress tolerance among pea genotypes, as already reported (El-Esawi et al. 2018; Khan et al. 2022). Correlating the gene expression and biochemical data, the highest number of significant correlations was detected in the two genotypes employed in Tunisia. In Merveille de Kelvedon, the relative expression of *PsSOD* was positively correlated to those of *PsCAT* and *PsAPX1*, suggesting a co-regulation of these scavenging genes. In Lincoln, the expression of *PsSOD* was also found to be significantly correlated with the expression of *PsCAT,* highlighting the co-regulation between these two functionally different genes. Additionally, always in Lincoln, a positive correlation between *PsSOD* expression level and the amount of H_2_O_2_ could suggest a potential relationship between the expression of this gene and oxidative stress, considering that SODs are able to catalyze the dismutation of the superoxide (O^−2^) radical into O^2^ and H_2_O_2_ (Bowler et al. 1994). A similar response to the treatments of these genes in both genotypes could be inferred. Nevertheless, looking at *PsSOD* and *PsCAT* expression on Merveille de Kelvedon and Lincoln inoculated plants, each pea genotype differentially responded to the two tested bacterial strains, with PG1 particularly affecting Lincoln stressed plants, while PG2 mainly impacted on Merveille de Kelvedon ones. In agreement with our data, a positive correlation between H_2_O_2_ level and proline content was previously found in plants subjected to environmental stress (Lee et al. 2022). The positive correlation between H_2_O_2_ and MDA in Merveille de Kelvedon could suggest a role of the peroxide in participating to lipid peroxidation and therefore to the membrane damages (Hajlaoui et al. 2009). Additionally, the correlation between MDA and proline in Merveille de Kelvedon could suggest that in presence of a cellular damage the plant adopts adaptive defensive mechanisms as osmoprotectants, as in celery under salt stress (Gao et al. 2023).

Chlorophyll a/b-binding proteins are reported to be involved in photosynthesis and to play a crucial role in capturing light energy (Bassi et al. 1997). The negative correlation between *PsCHL A/B* expression and the biochemical markers (MDA, proline, H_2_O_2_) in Merveille de Kelvedon, as well as between its expression and proline in Lincoln, suggested that when oxidative stress increases, and ROS rise, the expression of this gene decreases. This down-regulation may be a response to redirect cellular resources towards antioxidant defense mechanisms to cope with oxidative stress. It has been reported that reduced light absorption and increased anti-oxidative defense were associated with the down-regulation of chlorophyll a/b binding proteins (Munné-Bosch and Penuelas 2003; Xu et al. 2012). The down-regulation of *PsCHL A/B* in inoculated Merveille de Kelvedon and Lincoln plants under salt stress compared to uninoculated ones may also suggest that both PG1 and PG2 play a role in inducing this type of response. In Meraviglia d’Italia the only positive correlation was detected between *PsCAT* transcripts and proline; in upland drought tolerant rice varieties Lum et al. (2014) have already documented that their drought tolerance seemed to be correlated to activities of antioxidant enzymes, *e.g.*, catalase, and an increase of proline content. A similar response can be hypothesized for a salt stress condition. The reduced number of correlations found in Meraviglia d’Italia could suggest that other types of responses are activated (Almeida et al. 2013), and that it could be differently affected by the applied stress.

## Conclusion

The current study reveals that two non-pathogenic strains of *Erwinia* sp. are different in their PGP traits. By using an integrated approach, a picture of the pea plant status in three genotypes subjected to a salt stress condition was obtained and the role of the two bacterial considered *Erwinia* sp. strains has been highlighted. Results showed the relevance of plant genotype in determining the response to bacterial inoculants as well as the differences in the plant mechanisms activated to cope with the stress in the different plant/strain combination. Overall, this study emphasizes the importance of understanding the molecular and biochemical processes occurring in plant–microbe interactions at genotype level, and the influence on plant responses to environmental stresses. Further analyses are needed to clarify the behaviour of the three genotypes, such as the leaf water potential, and to verify the effects of the bacterial inoculation in field conditions, subjectd by an increased environmental unpredictability due to the climate change scenario.

### Supplementary Information

Below is the link to the electronic supplementary material.

Figure S1 Principal component analysis of biometric and physiological parameters (shoot height, node number, root and pod dry weight, chlorophyll content, and *g*_s_) performed with R (v 4.1.1) on the three genotypes (Merveille de Kelvedon, Lincoln and Meraviglia d’Italia) (n = 5 in Merveille de Kelvedon and Lincoln, while n = 10 in Meraviglia d’Italia. In (a), Principal component analysis (PCA) of samples; in (b), projection of variables, where angles are interpreted as correlations. The angle between two variable vectors represents the degree of correlation between them: adjacent (angle less than 90°) showed highly correlated variables, angle more than 90° showed uncorrelated ones. (PDF 24 kb)

Supplementary file 2 (DOCX 17 kb)

Supplementary file 3 (DOCX 24 kb)

## Data Availability

The datasets generated during and analyzed during the current study are available from the corresponding author on reasonable request.
