# Supplementary figures and images for: Impact of two Erwinia sp. on the response of diverse Pisum sativum genotypes under salt stress
Source: Physiol Mol Biol Plants. 2024 Feb 25;30(2):249–67. doi: 10.1007/s12298-024-01419-8 (PMC11016052; doi:10.1007/s12298-024-01419-8)

**(a)**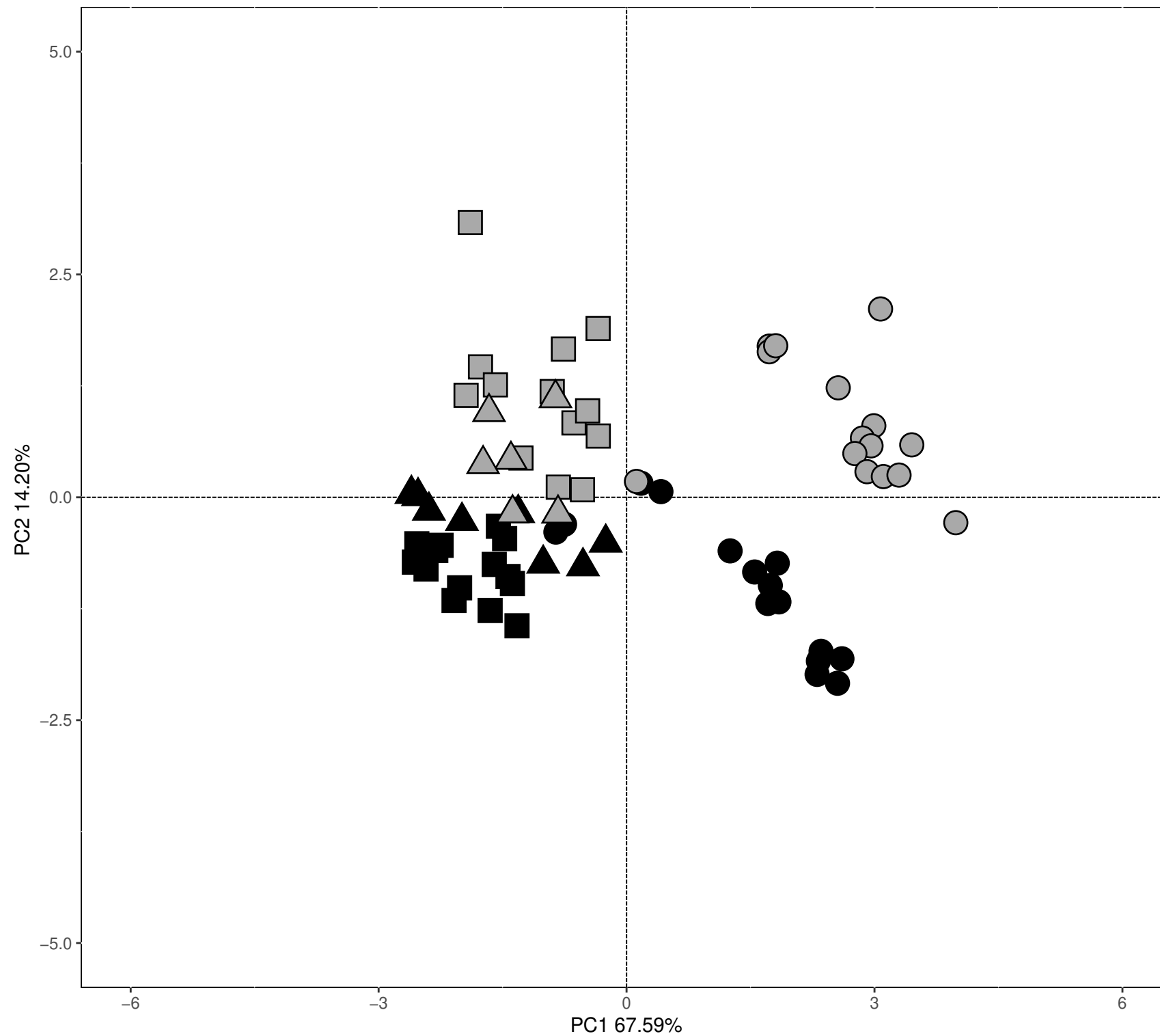**Genotype**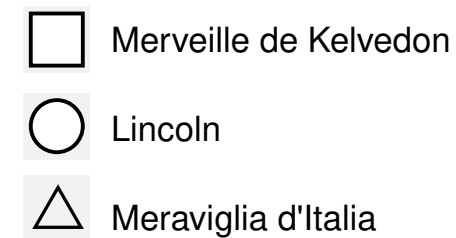**Condition**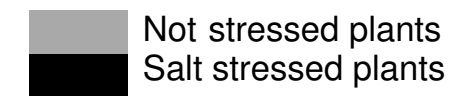**(b)****Variables – PCA**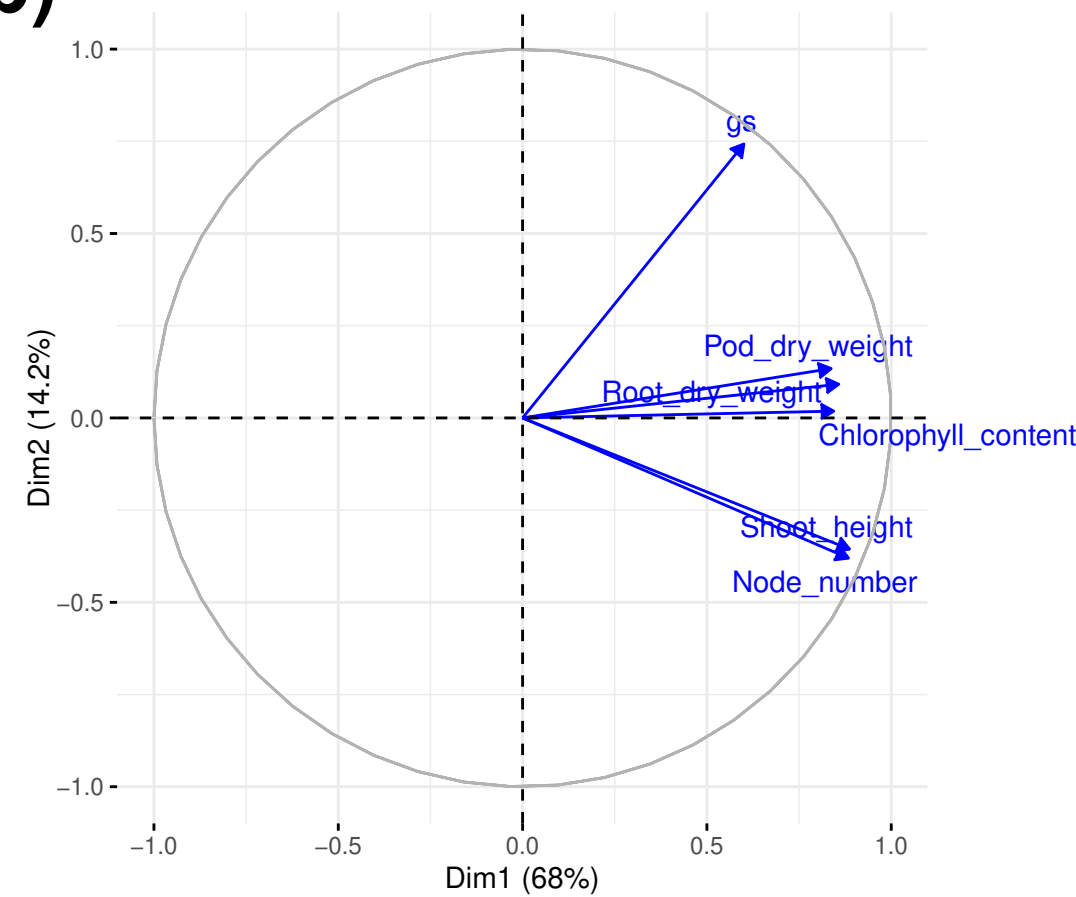

Supplement: Supplementary file 1 — Figure S1 Principal component analysis of biometric and physiological parameters (shoot height, node number, root and pod dry weight, chlorophyll content, and gs) performed with R (v 4.1.1) on the three genotypes (Merveille de Kelvedon, Lincoln and Meraviglia d’Italia) (n = 5 in Merveille de Kelvedon and Lincoln, while n = 10 in Meraviglia d’Italia. In (a), Principal component analysis (PCA) of samples; in (b), projection of variables, where angles are interpreted as correlations. The angle between two variable vectors represents the degree of correlation between them: adjacent (angle less than 90°) showed highly correlated variables, angle more than 90° showed uncorrelated ones. (PDF 24 kb) [file 12298_2024_1419_MOESM1_ESM.pdf]
