# Supplementary material for: Impact of two Erwinia sp. on the response of diverse Pisum sativum genotypes under salt stress
Source: Physiol Mol Biol Plants. 2024 Feb 25;30(2):249–67. doi: 10.1007/s12298-024-01419-8 (PMC11016052; doi:10.1007/s12298-024-01419-8)
Supplement: Supplementary file 2 — (DOCX 17 kb) [file 12298_2024_1419_MOESM2_ESM.docx]

**Supplementary Information**

**Molecular characterization of bacterial strains**

The mix consisted of: Hot Start PCR Master Mix (Thermo Fisher Scientific) (12.5 µL), 10 µM forward primer (0.5 µL), 10 µM reverse primer (0.5 µL), water (10.5 µL). PCR settings for 27-1495 amplification were the following: 2 min at 95°C; 30 s at 94°C, 30 s at 55°C, 90 s at 72°C (34 cycles); 7 min at 72°C. The obtained amplicons were checked on a 1 % agarose gel in 0.5%TAE buffer (20mM Tris-acetate, 0.5mM EDTA, 8.0 pH) stained with GelRed (VWR) and compared with the marker (100 bp, Invitrogen). After that, the PCR products were purified using the QIAquick® PCR Purification Kit (Qiagen) and sequenced at Ludwig Maximilian University Sequencing Service (Martinsried, Germany). The similarity of these sequences was compared using the NCBI online standard BASIC LOCAL ALIGNMENT SEARCH TOOL (BLASTN) algorithm (Altschul et al. 1990) on rRNA_typestrains/16S_ribosomal_RNA 16S ribosomal RNA (Bacteria and Archaea type strains). Using the software MEGA X (Kumar et al. 2018), a phylogenetic tree was then built. The settings for the phylogenetic tree were the following: statistical method, maximum likelihood; test of phylogeny, bootstrap method with 1000 re-samplings of the sequence alignment. A PCR was conducted to detect the presence of the *nifH* gene using 107F64 (TGYGAYCCSAARGCNGACTC) and 379R64 (GGCATNGCRAARCCRCCRCA) primer pair (Wang et al. 2022), using the same mix as above and the following program 94°C for 3 min, 94°C for 30 s, 60°C for 30 s, 72°C for 60 s (35 cycles), 72°C for 7 min. The obtained amplicons were checked on 1.5% agarose gel. *NifH* amplicon was purified and sequenced as written above and its similarity was checked on NCBI by BLASTN.

**Bacterial strains pathogenicity assay**

*Solanum lycopersicum* seeds (cultivar “San Marzano nano”, La Semiorto Sementi, Sarno, Italy) were sterilized in 2.5% v/v sodium hypochlorite for 20 min, then they were rinsed five times and placed in pots containing coconut fiber (watered with tap water) in a greenhouse. After nine days, the seedlings were transferred to pots (0.7 L) containing quartz sand, previously sterilized at 180°C for 3h, and watered with tap water. The plants were grown following the natural sun photoperiod in a greenhouse (January-February 2023). A first set of plants (with 5 replicates for each treatment) was inoculated with bacterial strains (PG1 and PG2) 16 days after sowing, or left uninoculated as control. A second set of tomato plants was inoculated 23 days after sowing. The plants were watered with tap water and monitored daily to evaluate potential symptoms onset. All the plants were harvested after 35 days after the sowing (20 and 13 days after the inoculation in the two sets, respectively), the root and shoot apparatus were visually checked for symptoms. Tomato plants inoculated with PG1 and PG2 at two different stages did not show any symptoms related to disease.

**Biochemical analysis of *P. sativum* stress markers**

Lipid peroxidation was determined from the measurement of malondialdehyde (MDA) content resulting from the thiobarbituric acid (TBA) reaction (Heath and Packer 1968), using an extinction coefficient of 155 mM^-1^ cm^-1^. In detail, MDA was assayed after digestion of leaf sample (~0.1 g) with 0.1 % (w/v) trichloroacetic acid (TCA) and centrifuged at 15000 g for 15 min at 4°C. Then 0.5 mL of supernatant with 1.5 mL 0.5% ΤΒΑ diluted in 20% TCA and incubated in water bath at 95°C for 25 min. The absorbance was read at 532 and 600 nm using a microplate reader (Infinite 200 PRO, TECAN, Switzerland).

Hydrogen peroxide (H_2_O_2_) was quantified using the KI method, as described by Loreto and Velikova (2001): 0.1 g of frozen leaves was homogenized with 0.1 % (w/v) trichloroacetic acid (TCA) and centrifuged at 15000 *g* for 15 min at 4°C. Then, the obtained supernatant (0.5 mL) was mixed with phosphate buffer 10 mM (0.5 mL) and potassium iodine 1M (1 mL) and the absorbance was read at 390 nm using a microplate reader (Tecan Infinite 200 PRO, TECAN, Switzerland). The H_2_O_2_ content was calculated based on the standard curve via different H_2_O_2_ concentrations (Loreto and Velikova 2001).

Free proline levels were determined using the ninhydrin reaction (Bates et al. 1973). In detail, for the proline assay, 0.1 g of frozen leaf sample was homogenized in 1.5 mL of 3% (w/v) aqueous sulfosalicylic acid and centrifuged at 12000 g, for 10 min at 4°C. Then 1 mL of supernatant was added to 1 mL acid-ninhydrin and 1 mL glacial acetic acid. The final solutions were incubated at 95°C for 1 h, then transferred to ice batch to stop the reaction. Finally, 2 mL toluene was added and vortexed for 20 sec and kept at room temperature for 30 min until separation of two phases. The absorbance of assay mixture was recorded at 520 nm using a microplate reader (Infinite 200 PRO, TECAN, Switzerland). Proline concentration was estimated from a D-proline standard curve (Bates et al. 1973).

**References**

Altschul SF, Gish W, Miller W, Myers EW, Lipman DJ (1990) Basic Local Alignment Search Tool. J Mol Biol 215:403-410. https://doi.org/10.1016/S0022-2836(05)80360-2

Bates LS, Waldren RP, Teare ID (1973) Rapid determination of free proline for water-stress studies. Plant Soil 39:205-207. https://doi.org/10.1007/BF00018060

Heath RL, Packer L (1968) Photoperoxidation in isolated chloroplasts: I. Kinetics and stoichiometry of fatty acid peroxidation. Arch Int Physiol Biochim Biophys 125:189-198. https://doi.org/10.1016/0003-9861(68)90654-1.

Kumar S, Stecher G, Li M, Knyaz C, Tamura K (2018) MEGA X: Molecular evolutionary genetics analysis across computing platforms. Mol Biol Evol 35:1547-1549. https://doi.org/10.1093/molbev/msy096

Loreto F, Velikova V (2001) Isoprene produced by leaves protects the photosynthetic apparatus against ozone damage, quenches ozone products, and reduces lipid peroxidation of cellular membranes. Plant Physiol 127:1781-1787. https://doi.org/10.1104/pp.010497

Wang Z, Feng K, Wei Z, et al. (2022) Evaluation and redesign of the primers for detecting nitrogen cycling genes in environments. Methods Ecol Evol 13: 1976-1989. https://doi.org/10.1111/2041-210X.13946
