# Supplementary material for: Impact of two Erwinia sp. on the response of diverse Pisum sativum genotypes under salt stress
Source: Physiol Mol Biol Plants. 2024 Feb 25;30(2):249–67. doi: 10.1007/s12298-024-01419-8 (PMC11016052; doi:10.1007/s12298-024-01419-8)
Supplement: Supplementary file 3 — (DOCX 24 kb) [file 12298_2024_1419_MOESM3_ESM.docx]

**Table S1** Results of the three-way ANOVA testing of the effects of the genotype, stress and type of inocula on biometric and physiological parameters. df = degree of freedom, F = F ratios, Sig = *p*-value and degree of significance (*** P < 0.001; ** P < 0.01; * P < 0.05)

| **Source** | df | Shoot height  (cm) | |  | Root dry weight  (g) | |  | Node number | |  | Pod dry weight  (g) | |  | Chlorophyll content  (°SPAD) | |  | g_s_  (mol m^-2^ s^-1^) | |  |
| --- | --- | --- | --- | --- | --- | --- | --- | --- | --- | --- | --- | --- | --- | --- | --- | --- | --- | --- | --- |
|  |  | F | Sig |  | F | Sig |  | F | Sig |  | F | Sig |  | F | Sig |  | F | Sig |  |
| Genotype | 2 | 64.194 | **<2e-16 ***** |  | 84.537 | **<2e-16 ***** |  | 48.114 | **2.3e-15 ***** |  | 204.502 | **< 2e-16 ***** |  | 61.129 | **< 2e-16 ***** |  | 46.827 | **4.45e-15 ***** |  |
| Stress | 1 | 6.679 | **0.0112 *** |  | 0.990 | 0.3222 |  | 2.164 | 0.144 |  | 1.957 | 0.16491 |  | 24.478 | **3.04e-06 ***** |  | 159.230 | **< 2e-16 ***** |  |
| Inocula | 2 | 0.696 | 0.5009 |  | 0.365 | 0.6948 |  | 1.067 | 0.348 |  | 0.197 | 0.82147 |  | 0.302 | 0.740045 |  | 0.649 | 0.525 |  |

**Table S2** Correlation matrix (based on Pearson correlation) among the seven variables used in the PCA, including biometric (shoot height, number of nodes, root and pod dry weight) and physiological parameters (chlorophyll content and g_s_) recorded in Merveille de Kelvedon and Lincoln pea genotypes. Asterisks showed the *p*-value of the correlation (***=p < 0.001).

|  | **Shoot height** | **Node number** | **Chlorophyll content** | **Root dry weight** | **Pod dry weight** | **g_s_** |
| --- | --- | --- | --- | --- | --- | --- |
| **Shoot height** | 1 | 0.89*** | 0.6 | 0.77*** | 0.74*** | 0.21 |
| **Node number** | 0.89*** | 1 | 0.53 | 0.63 | 0.64*** | 0.07 |
| **Chlorophyll content** | 0.6 | 0.53 | 1 | 0.38 | 0.45 | 0.53 |
| **Root dry weight** | 0.77*** | 0.63 | 0.38 | 1 | 0.84*** | 0.28 |
| **Pod dry weight** | 0.74*** | 0.64*** | 0.45 | 0.84*** | 1 | 0.31 |
| **g_s_** | 0.21 | 0.07 | 0.53 | 0.28 | 0.31 | 1 |

**Table S3** Biometric parameters (shoot height, number of nodes, root, pod and shoot dry weight) in Merveille de Kelvedon, Lincoln and Meraviglia d’Italia pea genotypes. All data are expressed as mean ± SD (standard deviation). No variables showed significant differences among treatments (*p*-value > 0.05) NS: not stressed plants; S: salt stressed plants; C: control, uninoculated plants

| **Genotype** | **Condition** | **Inoculation** | **Shoot height**  **(cm)** | **Node number** | **Number of pods** | **Root dry weight**  **(g)** | **Pod dry weight**  **(g)** | **Shoot dry weight**  **(g)** |
| --- | --- | --- | --- | --- | --- | --- | --- | --- |
| **Merveille de Kelvedon** | **NS** | **PG1** | 15.5±3.51 | 8.25±2.63 | 1±0 | 0.07±0.04 | 0.16±0.09 |  |
|  |  | **PG2** | 14.4±5.13 | 8.4±2.7 | 1±0 | 0.07±0.02 | 0.11±0.04 |  |
|  |  | **C** | 17±2.92 | 9.6±1.34 | 1±0 | 0.05±0.02 | 0.14±0.07 |  |
|  | **S** | **PG1** | 14.4±2.3 | 8.4±1.34 | 1±0 | 0.09±0.03 | 0.15±0.02 |  |
|  |  | **PG2** | 12.6±3.91 | 7.2±0.84 | 1±0 | 0.07±0.01 | 0.14±0.03 |  |
|  |  | **C** | 17.4±3.29 | 9.2±0.84 | 1±0 | 0.08±0.02 | 0.17±0.1 |  |
| **Lincoln** | **NS** | **PG1** | 28.2±2.39 | 11±1.22 | 1.8±0.84 | 0.17±0.05 | 0.49±0.28 |  |
|  |  | **PG2** | 33±3 | 12.8±1.1 | 1.4±0.55 | 0.22±0.05 | 0.74±0.11 |  |
|  |  | **C** | 33.6±2.61 | 12.4±0.55 | 1.4±0.55 | 0.21±0.08 | 0.59±0.24 |  |
|  | **S** | **PG1** | 31.4±4.1 | 13.2±1.48 | 1.2±0.45 | 0.15±0.05 | 0.51±0.21 |  |
|  |  | **PG2** | 31.4±4.93 | 11.6±1.67 | 1.6±0.55 | 0.15±0.08 | 0.34±0.23 |  |
|  |  | **C** | 28.6±5.81 | 12.2±1.92 | 1.4±0.55 | 0.16±0.07 | 0.58±0.19 |  |
| **Meraviglia d’Italia** | **NS** | **PG1** | 21±3.78 | 7.63±1.41 | 1.25±0.35 | 0.09±0.02 | 0.06±0.03 | 0.3±0.05 |
|  |  | **PG2** | 20.63±1.83 | 7.75±0.52 | 2±0 | 0.07±0.01 | 0.08±0 | 0.26±0.15 |
|  |  | **C** | 21±5.72 | 8.3±1.64 | 1±0 | 0.07±0.01 | 0.07±0.07 | 0.22±0.03 |
|  | **S** | **PG1** | 18.71±2.21 | 7.86±0.9 | 1.5±0.45 | 0.1±0.02 | 0.05±0.05 | 0.37±0.16 |
|  |  | **PG2** | 17.25±1.83 | 6.63±0.52 | 1±0.5 | 0.07±0.01 | 0.03±0.02 | 0.28±0.12 |
|  |  | **C** | 17.75±5.06 | 8.13±1.96 | 1.25±0.5 | 0.08±0.04 | 0.08±0.06 | 0.26±0.11 |

**Table S4** Results of the three-way ANOVA testing of the effects of the genotype, stress and type of inocula on biochemical data. df = degree of freedom, F = F ratios, Sig = *p*-value and degree of significance ( *** P < 0.001; ** P < 0.01; * P < 0.05)

| **Source** | df | MDA | |  | H2O2 | |  | Proline | |
| --- | --- | --- | --- | --- | --- | --- | --- | --- | --- |
|  |  | F | Sig |  | F | Sig |  | F | Sig |
| Genotype | 2 | 124.661 | **<2e-16 ***** |  | 193.052 | **<2e-16 ***** |  | 12027 | **< 2e-16 ***** |
| Stress | 1 | 13.983 | **0.000345 ***** |  | 45.868 | **3.63e-09 ***** |  | 50098 | **< 2e-16 ***** |
| Inocula | 2 | 6.004 | **0.003720 **** |  | 8.953 | **0.000354 ***** |  | 7460 | **< 2e-16 ***** |
